# Development of a Tool to Predict Outcome of Autologous Chondrocyte Implantation

**DOI:** 10.1177/1947603516650002

**Published:** 2016-05-23

**Authors:** M. Naomi Dugard, Jan Herman Kuiper, Jane Parker, Sally Roberts, Eric Robinson, Paul Harrison, James B. Richardson

**Affiliations:** 1 Robert Jones & Agnes Hunt Orthopaedic NHS Foundation Trust Hospital, Oswestry, Shropshire, UK; 2 Institute of Science and Technology in Medicine, Keele University, Keele, Staffordshire, UK

**Keywords:** prediction tool, success of ACI, further surgery, cartilage repair

## Abstract

**Objective:**

The study had 2 objectives: first, to evaluate the success of autologous chondrocyte implantation (ACI) in terms of incidence of surgical re-intervention, including arthroplasty, and investigate predictors of successful treatment outcome. The second objective was to derive a tool predicting a patient’s arthroplasty risk following ACI.

**Design:**

In this Level II, prognostic study, 170 ACI-treated patients (110 males [aged 36.8 ± 9.4 years]; 60 females [aged 38.1 ± 10.2 years]) completed a questionnaire about further surgery on their knee treated with ACI 10.9 ± 3.5 years previously. Factors commonly assessed preoperatively (age, gender, defect location and number, previous surgery at this site, and the preoperative Lysholm score) were used as independent factors in regression analyses.

**Results:**

At final follow-up (maximum of 19 years post-ACI), 40 patients (23.5%) had undergone surgical re-intervention following ACI. Twenty-six patients (15.3%) underwent arthroplasty, more commonly females (25%) than males (10%; *P* = 0.001). Cox regression analyses identified 4 factors associated with re-intervention: age at ACI, multiple operations before ACI, patellar defects, and lower pretreatment Lysholm scores (Nagelkerke’s *R*^2^ = 0.20). Six predictive items associated with risk of arthroplasty following ACI (Nagelkerke’s *R*^2^ = 0.34) were used to develop the Oswestry Risk of Knee Arthroplasty index with internal cross-validation.

**Conclusion:**

In a single-center study, we have identified 6 factors (age, gender, location and number of defects, number of previous operations, and Lysholm score before ACI) that appear to influence the likelihood of ACI patients progressing to arthroplasty. We have used this information to propose a formula or “tool” that could aid treatment decisions and improve patient selection for ACI.

## Introduction

Autologous chondrocyte implantation (ACI), first reported in 1994,^1^ is a cell therapy option for the treatment of focal cartilage defects in articular joints, particularly the knee.^2^ Although mid- to long-term durability has been demonstrated with this technique,^3-7^ current recommendations from the National Institute for Health and Care Excellence^8^ in the United Kingdom state that patients should be fully informed of the uncertainties about the long-term effectiveness of ACI. Since not all patients benefit from ACI, recognizing and understanding why the treatment was ultimately unsuccessful in these patients could influence who is selected for the procedure in the future. This would prevent subjecting these patients to an unnecessary treatment and hopefully help the search for a better treatment for this group. Treatment failure after ACI, however, has been inconsistently defined in studies,^9^ with various endpoints being used to define it. Loss of benefit in terms of clinical improvement, surgical re-intervention, or revision surgery are often used to evaluate risk factors for failure of joint reconstruction therapies.^10-12^ Total joint replacement has been suggested as a primary outcome for randomized clinical trials of disease modifying osteoarthritis drugs because it represents failure of the “organ,”^13-15^ although this can be viewed as heavily influenced by the treating surgeon. Even recognizing that joint replacements can last several decades, patients having arthroplasty below the age of 50 are likely to outlast their implant and may need revision.^16^ This is a more complicated procedure, with the revision prosthesis often less effective, and has cost-benefit implications, both for the patient and health care provider.

We therefore investigated the clinical outcome in a large cohort of patients treated with ACI, identifying factors associated with arthroplasty. Our overall aim was to develop an internally validated scoring system that could be used to aid the treatment decision-making process to better match the most appropriate treatment to each individual patient. In this study, we have determined the incidence of surgical re-intervention after ACI treatment in our center, investigated which factors were associated with a return of the functional outcome to its baseline value, or the requirement for further surgery after the treatment and, from this, developed a predictive tool to aid clinical decision making when considering ACI surgery.

## Materials and Methods

Study participants consisted of a cohort of consecutive patients treated in our center with ACI (between 1996 and 2010) for cartilage defects of the knee joint. Ethical approval for this retrospective study was obtained from the South Staffordshire Local Research Ethics Committee (Reference 09/H1203/90), and patients, who had given their informed consent to the research study, were approached at least 12 months after receiving an ACI and asked to complete a postal questionnaire. Follow-up time was calculated from the date of cell implantation in ACI to the date of questionnaire return. Clinical scores and details including indications for further surgery and arthroplasty were verified using hospital patient records. Patients treated with ACI for the presence of one or more grades II to IV chondral or osteochondral defects (Outerbridge classification^17^ or International Cartilage Repair Society score^18^) in their affected knee were identified using hospital records. Baseline measurements, features of defects, and co-incidental treatments were collected retrospectively using patient medical files. The ACI procedure was performed in 2 stages as previously described.^1,6^ Briefly, chondrocytes were isolated from a small portion of full thickness cartilage harvested at arthroscopy (stage 1), and their numbers expanded in monolayer culture.^19^ An arthrotomy was performed 3 weeks later (stage 2), during which the defect edges were cut back to healthy cartilage. Defect details were recorded on a knee diagram^20^ before the defect(s) were covered by periosteum (from the proximal medial tibia) or collagen membrane (Chondro-Gide; Pharma AG, Wolhusen, Switzerland). A suspension of the cultured chondrocytes in autologous serum was injected beneath the patch.

Immediately after ACI surgery, patients were provided with a rehabilitation protocol tailored to the site of the treated defect, outlining the appropriate level of motion and weight-bearing exercise to follow.^21^ The Lysholm score, a recommended patient-reported outcome measure for the knee,^22-24^ was used to assess improvement in clinical outcome. One year following ACI, an arthroscopic procedure was offered to all patients as part of routine clinical follow-up, during which hypertrophic cartilage growth, if present in the treated area, was debrided. This arthroscopy was not recorded as a re-intervention.

Failure of ACI was investigated based on 3 definitions: (1) the occurrence of knee arthroplasty, (2) the occurrence of any further surgery other than the routine follow-up arthroscopy at 1 year, and (3) the return of the functional outcome (Lysholm score) to its baseline value or below once past the 1-year post-ACI point.

Normally distributed continuous data (checked using Q-Q plots) were expressed in terms of mean ± standard deviation (SD), or otherwise expressed as median and interquartile range (IQR). Associations between categorical variables were investigated using the chi-squared test. Preoperative and annual postoperative scores were compared using a Wilcoxon signed-rank test for paired samples (e.g., baseline vs. 1-year scores) and a Mann-Whitney U test for independent samples (e.g., female vs. male patients). A Kaplan-Meier survival analysis was used to determine the cumulative risk of failure (arthroplasty, further surgery, or return to baseline Lysholm score) over time; cases that did not fail were censored at the end of their observation period. Differences between genders were tested using the log-rank test. Continuous and categorical predictors of the risk of return of functional outcome to baseline value, re-intervention, or arthroplasty during the follow-up time were assessed using Cox proportional hazard models. The quality of the various predictors and predictive models was assessed using a generalization of the coefficient of determination (Nagelkerke’s R2).^25,26^ This coefficient captures aspects of calibration (the agreement between predicted and observed outcome) and discrimination (how well the score distinguishes between cases who do and do not fail),^27^ both of which are important in prognostic risk scores.^28^

The proportional hazard assumption was validated for each predictor identified by univariable Cox regression (with P < 0.15); the best-performing individual items were included into a multivariable Cox proportional hazard model. Model parameters were further optimized with a penalized regression method that used a shrinkage procedure based on leave-one-out cross-validation.^29^ The cross-validation step served as an internal validation of the prognostic model by repeatedly splitting the sample in a derivation sample, used to produce the model, and a validation sample, used to test the model. The shrinkage procedure automatically prevented “overfitting” by reducing (shrinking) the coefficient value for predictors in the original model that failed to predict the correct outcome in the validation step.^26^ The result was an internally validated prognostic model.^26^

Statistical analyses were performed using SPSS version 19.0 (SPSS Science Inc., Chicago, IL) and R version 2.13.0 (R Foundation for Statistical Computing) with the “survival” and “penalized” packages. A 2-tailed *P* value of <0.05 was assumed to denote statistical significance; a listwise deletion approach was performed to handle missing data.

## Results

A study questionnaire was sent to 202 knee patients treated with ACI in our center; 170 completed and returned it, corresponding to a response rate of 84%. The study cohort consisted of 110 men and 60 women, with a mean age at ACI treatment of 36.8 ± 9.4 and 38.1 ± 10.2 years, respectively (P = 0.42); 6 surgeons had carried out the ACI procedures, with 83% being performed by one surgeon alone. The mean follow-up time for these patients was 10.9 ± 3.5 years post-ACI (range = 4.6-18.6; **Table 1**). Twelve patients had no previous surgery of the knee before ACI, while 73 had one and 77 had multiple surgical procedures (up to a maximum of 11) on their knee prior to their ACI; details of previous surgery were unavailable for 8 patients.

**Table 1.** Patient Demographics Subdivided Into Patients With and Without Revision Surgery Post-ACI Treatment.

Patient Characteristics	Total Patients (*N* = 170)	Patients Receiving Further Surgery	
		Yes (*n* = 40)	No (*n* = 130)
Male–female	110:60	21:19	89:41
Age at ACI (years), mean ± SD [range]	37.3 ± 9.7 [15.1-65.8]	40.3 ± 10.4 [18.7-65.6]	36.3 ± 9.3 [15.1-65.8]
Follow-up time (years), mean ± SD [range]	10.9 ± 3.5 [4.6-18.6]	11.9 ± 2.9 [5.8-16.7]	10.6 ± 3.6 [4.6-18.6]
Age at follow-up (years), mean ± SD [range]	48.1 ± 10.2 [23.0-77.0]	52.1 ± 10.4 [33.0-77.0]	46.9 ± 9.8 [23.0-70.9]
Patients with single defects	124	26	98
Size of single defect (cm^2^), median [IQR]	4.0 [2.4-6.0]	4.2 [2.3-6.8]	3.7 [2.5-5.5]
Anatomical location of single defect			
Medial femoral condyle	74	14	60
Lateral femoral condyle	28	6	22
Patella	10	4	6
Trochlea	8	2	6
Lateral tibial plateau	3	1	2
Medial tibial plateau	1	1	0
Patients with multiple defects	46	14	32
Previous operations [yes–no] (n)	151:12 (163)	37:2 (39)	114:10 (124)
Patients with co-incidental surgery [yes:no]	100:70	25:15	75:55

Normally distributed data summarized by mean ± standard deviation [range], and non-normally distributed data summarized by median (interquartile range [IQR]). ACI = autologous chondrocyte implantation.

**Figure 1.**
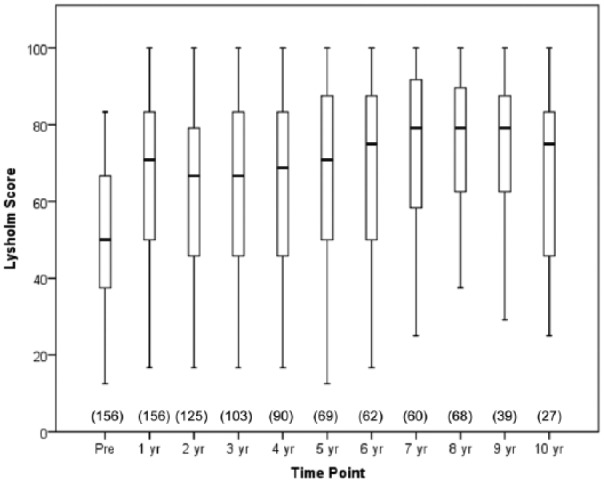
Boxplot with whiskers of the preoperative (Pre) and annual Lysholm scores of all patients up to 10 years after ACI treatment. For those patients who underwent arthroplasty, no further Lysholm scores were recorded after this procedure. The central box spans the first quartile to the third quartile (the interquartile range), the segment inside shows the median, and the “whiskers” above and below the box show the locations of the minimum and maximum. Patient numbers for each time point are in parentheses.

Changes in the clinical outcome (Lysholm score) of the total patient population are shown in **Figure 1**. The median Lysholm score improved for the whole patient group from baseline to 1 year posttreatment (Wilcoxon signed-rank-test, P < 0.001) and improvement was sustained for a further 10 years post-ACI (Fig. 1); 72% (103/142) of patients were improved, 18% (25/142) had lower scores, and 10% (14/142) showed no improvement. Lysholm scores were significantly higher in males (75.0 [IQR = 50.0-83.3] than females (56.3 [IQR = 41.7-79.2]; Mann-Whitney U test, P = 0.01) 1 year post-ACI, although the pre-operative scores were not significantly different (male [54.2; IQR = 37.5-66.7] vs. female [43.8; IQR = 37.5-54.2], Mann-Whitney U test, P = 0.062).

In all, 40 (23.5%) patients had undergone a further surgical procedure in the same knee compartment previously treated with ACI (**Table 2**). At 5 years, the cumulative risk of a further operation was 16%, increasing to 24% at 10 years. When using the return of functional outcome (Lysholm score) to its baseline value or below as an indication of failure, 43% of patients had returned to baseline at 5 years, 50% of patients at 8 years, and 61% of patients at 10 years following ACI.

**Table 2.** Types of Further Surgeries Performed during the Follow-Up Period in 40 Patients.

Surgery	No. of Patients
Realignment	5
Autologous chondrocyte implantation	4
Chondroplasty	1
Mosaicplasty	2
Arthroplasty (unicompartmental or total)	26

**Figure 2.**
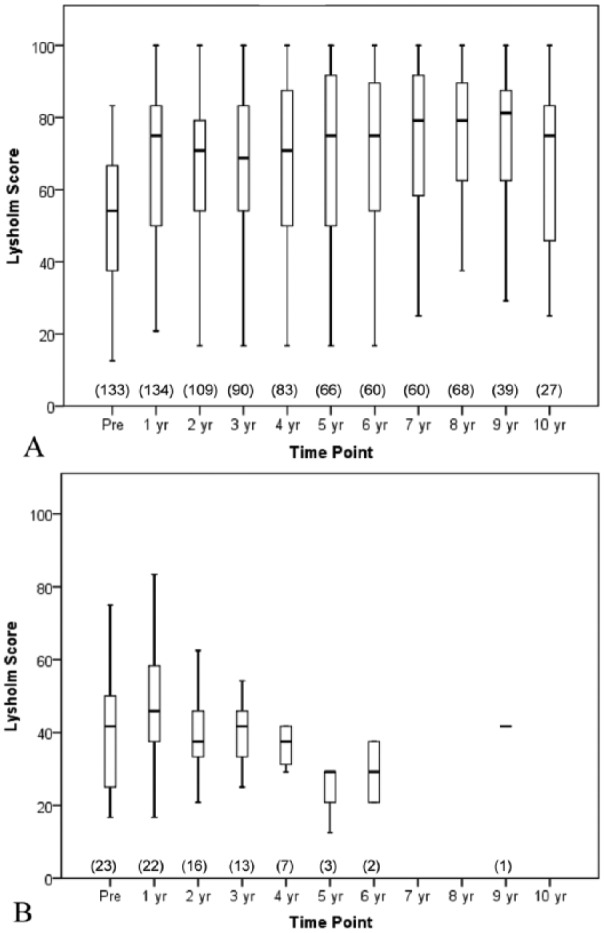
Boxplot with whiskers of the preoperative (Pre) and annual Lysholm scores following autologous chondrocyte implantation treatment in patients who did not progress to arthroplasty (**A**) and those who did (**B**). No further Lysholm scores were recorded after patients had undergone arthroplasty. The central box spans the first quartile to the third quartile (the interquartile range), the segment inside shows the median, and the “whiskers” above and below the box show the locations of the minimum and maximum. Patient numbers for each time point are in parentheses.

**Figure 3.**
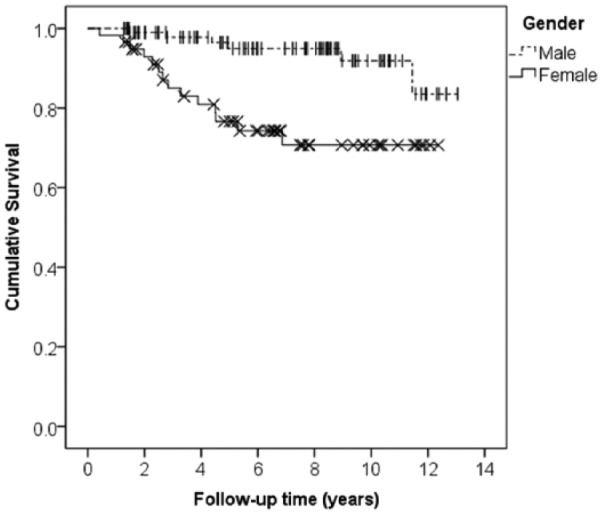
Kaplan-Meier survival analysis for all 170 patients receiving autologous chondrocyte implantation stratified by gender. Treatment failure, as defined by arthroplasty, was recorded in 26 patients (| and × show final follow-up points for individual patients [i.e., “censored” data], for males and females, respectively).

Twenty-six patients (15%) underwent knee arthroplasty 4.4 ± 2.6 (range = 0.4-11.4) years after ACI. Lysholm scores for this patient group are shown in **Figure 2**; they recorded significantly lower pre-ACI Lysholm scores than those patients who did not have arthroplasty (41.7 [IQR = 25.0-54.2] vs. 54.2 [IQR = 37.5-66.7], P < 0.005). While both groups demonstrated improvement in knee function at 1 year post-ACI, this was greater and significant for those who did not progress to arthroplasty (Lysholm score increasing to 75.0 points [IQR = 50.0-83.3; P < 0.001; Fig. 2A), compared to the lesser and not-significant increase seen for those who went on to have an arthroplasty (12 month Lysholm of 45.8 [IQR = 35.4-59.4; P = 0.181; Fig. 2B). The cumulative probability of remaining free from arthroplasty at 5 years was 90% (95% in male and 76% in female patients) and at 10 years was 84% (92% in males and 71% in females), corresponding to a significant difference in hazard rates between males and females (*P* < 0.001, log-rank test; **Figure 3**).

Significant individual categorical and continuous factors, identified using univariate Cox regression analyses, which influenced the likelihood of further surgery, were age at ACI, previous operations (none or single = 0, multiple = 1), and preoperative Lysholm score (**Table 3**). Factors increasing the risk that clinical function would return to that recorded at baseline were having undergone a previous operation and a higher baseline Lysholm score (**Table 4**), while those influencing the likelihood of arthroplasty after ACI were age at ACI, gender (male = 0, female = 1), defect number, defects (single = 0, multiple = 1), patellar defect (no = 0, yes = 1), previous operations (none or single = 0, multiple = 1), and preoperative Lysholm score (**Table 5**). Multivariable analyses were then used to determine which combined predictors were associated with further surgery and arthroplasty after ACI. Age, patellar defects, multiple operations before ACI, and preoperative Lysholm score were, in combination, significantly associated with an increased risk of further surgery in the final multivariable model (Nagelkerke’s R2 = 0.20; **Table 6**). Likewise, more defects, having had multiple previous operations, undergoing any parallel intervention, and a higher baseline Lysholm score were significantly associated with an increased risk that clinical function would return to baseline levels (Nagelkerke’s R2 = 0.18; **Table 7**). Finally, age, female gender, patellar defects, defect number, multiple operations before ACI, and preoperative Lysholm score were significantly associated with an increased risk of arthroplasty in the final multivariable model (Nagelkerke’s R2 = 0.34; **Table 8**).

**Table 3.** Univariable Analysis of Influence from Categorical and Continuous Variables on the Survival of ACI Using Any Further Surgical Treatment as the Endpoint.

Variable	*P* Value	Hazard Ratio (95% CI)	Nagelkerke’s *R*^2^
Age at ACI	0.013*	1.04 (1.01-1.08)	0.038
Gender (male = 0, female = 1)	0.057	1.83 (0.98-3.40	0.023
Defect number	0.058	1.61 (0.98-2.10)	0.021
Defects (single = 0, multiple = 1)	0.185	1.55 (0.81-2.98)	0.011
Maximum defect grade	0.717	1.11 (0.63-1.97)	0.001
Patch type (collagen = 0, periosteum = 1)	0.442	1.29 (0.67-2.48)	0.004
MFC defect (no = 0, yes = 1)	0.560	0.83 (0.45-1.55)	0.002
LFC defect (no = 0, yes = 1)	0.773	0.90 (0.43-1.88)	0.0006
Patellar defect (no = 0, yes = 1)	0.011*	2.55 (1.24-5.23)	0.035
Trochlear defect (no = 0, yes = 1)	0.573	0.78 (0.33-1.86)	0.002
MTP defect (no = 0, yes = 1)	0.340	1.65 (0.59-4.65)	0.005
LTP defect (no = 0, yes = 1)	0.411	1.64 (0.51-5.32)	0.004
Previous operations (no = 0, yes = 1)	0.524	1.59 (0.38-6.59)	0.003
Previous operations (none or single = 0, multiple = 1)	0.027*	2.10(1.09-4.03)	0.034
Previous microfracture (no = 0, yes = 1)	0.852	0.93 (0.43-2.02)	0.0002
Previous cartilage regeneration^a^ (no = 0, yes = 1)	0.337	1.39 (0.71-2.70)	0.006
Parallel operation (no = 0, yes = 1)	0.742	0.90 (0.47-1.70)	0.0007
Parallel osteotomy (no = 0, yes = 1)	0.180	3.42 (1.34-8.74)	0.003
Parallel patella realignment (no = 0, yes = 1)	0.686	0.66 (0.09-4.85)	0.001
Parallel meniscal surgery (no = 0, yes = 1)	0.796	0.87 (0.31-2.45)	0.0005
Preoperative Lysholm score	0.001*	0.97 (0.95-0.99)	0.078

Calculation of *P* values, hazard ratios, and Nagelkerke’s *R*^2^ for each separate term determined from a univariable Cox model with that term. ACI = autologous chondrocyte implantation; CI = confidence interval; MFC = medial femoral condyle; LFC = lateral femoral condyle; MTP = medial tibial plateau; LTP = lateral tibial plateau.

a Cartilage regeneration techniques included ACI, mosaicplasty, microfracture.

* Factor significantly influences risk of further surgery (*P* < 0.05).

**Table 4.** Univariable Analysis of Influence from Categorical and Continuous Variables on the Survival of ACI Using the Return of the Functional Outcome (Lysholm score) to Its Baseline Value or Below as the Endpoint.

Variable	*P* Value	Hazard Ratio (95% CI)	Nagelkerke’s *R*^2^
Age at ACI	0.38	1.01 (0.99-1.03)	0.005
Gender (male = 0, female = 1)	0.98	1.01 (0.68-1.50	0.000
Defect number	0.089	1.33 (0.96-1.85)	0.016
Defects (single = 0, multiple = 1)	0.21	1.31 (0.86-1.99)	0.009
Maximum defect grade	0.75	0.94 (0.64-1.37)	0.001
Patch type (collagen = 0, periosteum = 1)	0.57	0.89 (0.59-1.34)	0.002
MFC defect (no = 0, yes = 1)	0.72	0.93 (0.63-1.37)	0.001
LFC defect (no = 0, yes = 1)	0.70	0.91 (0.58-1.46)	0.001
Patellar defect (no = 0, yes = 1)	0.055	1.65 (0.99-2.76)	0.019
Trochlear defect (no = 0, yes = 1)	0.41	0.81 (0.49-1.34)	0.004
MTP defect (no = 0, yes = 1)	0.39	1.38 (0.67-2.85)	0.004
LTP defect (no = 0, yes = 1)	0.080	1.99 (0.92-4.31)	0.015
Previous operations (no = 0, yes = 1)	0.028*	2.77 (1.12-6.88)	0.039
Previous operations (none or single =0, multiple = 1)	0.018*	1.46 (1.07-2.00)	0.036
Previous microfracture (no = 0, yes = 1)	0.33	0.77 (0.46-1.29)	0.006
Previous cartilage regeneration^a^ (no = 0, yes = 1)	0.38	1.00 (0.999-1.001)	0.004
Parallel operation (no = 0, yes = 1)	0.11	1.37 (0.93-2.02)	0.015
Parallel osteotomy (no = 0, yes = 1)	0.64	1.22 (0.53-2.80)	0.001
Parallel patella realignment (no = 0, yes = 1)	0.46	1.41 (0.57-3.48)	0.003
Parallel meniscal surgery (no = 0, yes = 1)	0.78	1.09 (0.61-1.95)	0.000
Preoperative Lysholm score	0.004*	1.02 (1.01-1.03)	0.054

Calculation of *P* values, hazard ratios, and Nagelkerke’s *R*^2^ for each separate term determined from a univariable Cox model with that term. ACI = autologous chondrocyte implantation; CI = confidence interval; MFC = medial femoral condyle; LFC = lateral femoral condyle; MTP = medial tibial plateau; LTP = lateral tibial plateau.

a Cartilage regeneration techniques included ACI, mosaicplasty, microfracture.

* Factor significantly influences risk of further surgery (*P* < 0.05).

**Table 5.** Univariable Analysis of Influence from Categorical and Continuous Variables on the Survival of ACI Using Arthroplasty as the Endpoint.

Variable	*P* Value	Hazard Ratio (95% CI)	Nagelkerke’s *R*^2^
Age at ACI	0.000*	1.08 (1.04-1.12)	0.101
Gender (male = 0, female = 1)	0.009*	2.84 (1.30-6.19	0.051
Defect number	0.000*	2.85 (1.69-4.81)	0.092
Defects (single = 0, multiple = 1)	0.001*	3.64 (1.68-7.89)	0.076
Maximum defect grade	0.346	1.43 (0.68-3.03)	0.009
Patch type (collagen = 0, periosteum = 1)	0.451	0.74 (0.34-1.62)	0.004
MFC defect (no = 0, yes = 1)	0.570	0.80 (0.37-1.73)	0.002
LFC defect (no = 0, yes = 1)	0.734	1.16 (0.49-2.77)	0.0008
Patellar defect (no = 0, yes = 1)	0.001*	4.22 (1.87-9.53)	0.072
Trochlear defect (no = 0, yes = 1)	0.827	1.12 (0.42-2.96)	0.0003
MTP defect (no = 0, yes = 1)	0.293	1.91 (0.57-6.36)	0.007
LTP defect (no = 0, yes = 1)	0.539	1.57 (0.37-6.65)	0.0025
Previous operations (no = 0, yes = 1)	0.908	0.918 (0.22-3.89)	0.0001
Previous operations (none or single = 0, multiple = 1)	0.014*	2.99 (1. 25-7.16)	0.067
Previous microfracture (no = 0, yes = 1)	0.450	0.66 (0.23-1.93)	0.0049
Previous cartilage regeneration^a^ (no = 0, yes = 1)	0.902	1.00 (0.99-1.00)	0.0001
Parallel operation (no = 0, yes = 1)	0.753	1.13 (0.52-2.47)	0.0007
Parallel osteotomy (no = 0, yes = 1)	0.190	4.13 (1.42-12.01)	0.0036
Parallel patella realignment (no = 0, yes = 1)	0.90	1.14 (0.15-8.49)	0.0001
Parallel meniscal surgery (no = 0, yes = 1)	0.94	1.05 (0.32-3.49)	<0.0001
Preoperative Lysholm score	0.007*	0.97 (0.95-0.99)	0.059

Calculation of *P* values, hazard ratios, and Nagelkerke’s *R*^2^ for each separate term determined from a univariable Cox model with that term. ACI = autologous chondrocyte implantation; CI = confidence interval; MFC = medial femoral condyle; LFC = lateral femoral condyle; MTP = medial tibial plateau; LTP = lateral tibial plateau.

a Cartilage regeneration techniques included ACI, mosaicplasty, microfracture.

* Factor significantly influences risk of arthroplasty (*P* < 0.05).

**Table 6.** Multivariable Analysis of the Influence of Preoperative Lysholm Score and Other Categorical and Continuous Parameters on the Survival of ACI Using Further Surgical Treatment as the Endpoint.

Variable	Coefficient	*P* Value	Hazard Ratio (95% CI)
Full model^a^			
Age at ACI	0.04	0.099	1.04 (0.99-1.08)
Gender (male = 0, female = 1)	0.55	0.121	1.74 (0.87-3.49)
Defect number	0.45	0.128	1.57 (0.88-2.80)
Patellar defect (no = 0, yes = 1)	1.10	0.011*	2.99 (1.29-6.94)
Previous operations (none or single = 0, multiple = 1)	1.01	0.011*	2.75 (1.27-5.97)
Pre-ACI Lysholm score	−0.03	0.003*	0.97 (0.95-0.99)
Final model^b^			
Age at ACI	0.05	0.025*	1.05 (1.01-1.09)
Patellar defect (no = 0, yes = 1)	1.19	0.003*	3.29 (1.48-7.30)
Previous operations (none or single = 0, multiple = 1)	1.01	0.008*	2.75 (1.30-5.79)
Pre-ACI Lysholm score	−0.03	0.002*	0.97 (0.95-0.99)

Calculation of *P* values and hazard ratios based on full Cox model (using all univariables with *P* < 0.15). ACI = autologous chondrocyte implantation; CI = confidence interval.

a Nagelkerke’s *R*^2^ = 0.226 for the full model.

b Nagelkerke’s *R*^2^ = 0.202 for the final model.

* Factor significantly influences risk of further surgery after ACI (*P* < 0.05).

**Table 7.** Multivariable Analysis of the Influence of Preoperative Lysholm Score and Other Categorical and Continuous Parameters on the Survival of ACI Using the Return of the Functional Outcome (Lysholm Score) to Its Baseline Value or Below as the Endpoint.

Variable	Coefficient	*P* Value	Hazard Ratio (95% CI)
Full model^a^			
Defect number	0.37	0.064	1.44 (0.98-2.12)
Patellar defect (no = 0, yes = 1)	0.51	0.11	1.67 (0.89-3.11)
LTP defect (no = 0, yes = 1)	0.24	0.60	1.27 (0.52-3.09)
Previous operations (none or single = 0, multiple = 1)	0.62	<0.001*	1.85 (1.29-2.65)
Parallel operation (no = 0, yes = 1)	0.33	0.18	1.39 (0.86-2.26)
Pre-ACI Lysholm score	0.025	<0.001*	1.03 (1.02-1.04)
Final model^b^			
Defect number	0.46	0.012*	1.58 (1.11-2.26)
Previous operations (none or single = 0, multiple = 1)	0.57	<0.001*	1.77 (1.26-2.48)
Parallel operation (no = 0, yes = 1)	0.44	0.049*	1.55 (1.00-2.40)
Pre-ACI Lysholm score	0.024	<0.001*	1.03 (1.01-1.04)

Calculation of *P* values and hazard ratios based on full Cox model (based on all univariables with *P* < 0.15). ACI = autologous chondrocyte implantation; CI = confidence interval. LTP = lateral tibial plateau.

a Nagelkerke’s *R*^2^ = 0.188 for the full model.

b Nagelkerke’s *R*^2^ = 0.175 for the final model.

* Factor significantly influences risk of further surgery after ACI (*P* < 0.05).

**Table 8.** Multivariable Analysis of the Influence of Preoperative Lysholm Score and Other Categorical and Continuous Parameters on the Survival of ACI Using Knee Arthroplasty as the Endpoint.

Variable	Coefficient	*P* Value	Hazard Ratio (95% CI)
Full model^a^			
Age at ACI	0.07	0.007*	1.07 (1.02-1.12)
Gender (male = 0, female = 1)	1.05	0.017*	2.85 (1.20-6.75)
Defect number	1.57	0.020*	4.83 (1.28-18.24)
Defects (single = 0, multiple = 1)	−0.89	0.352	0.41 (0.06-2.67)
Patellar defect (no = 0, yes = 1)	1.68	0.002*	5.38 (1.89-15.29)
Previous operations (none or single = 0, multiple = 1)	1.23	0.020*	3.42 (1.22-9.64)
Pre-ACI Lysholm score	−0.03	0.017*	0.97 (0.94-0.99)
Final model^b^			
Age at ACI	0.07	0.009*	1.07 (1.02-1.12)
Gender (male = 0, female = 1)	1.00	0.021*	2.71 (1.164-6.31)
Defect number	1.01	0.002*	2.73 (1.45-5.15)
Patellar defect (no = 0, yes = 1)	1.66	0.002*	5.26 (1.87-14.79)
Previous operations (none or single = 0, multiple = 1)	1.32	0.011*	3.75 (1.36-10.38)
Pre-ACI Lysholm score	−0.04	0.018*	0.966 (0.94-0.99)

Calculation of *P* values and hazard ratios based on full Cox model (using all univariables with *P* < 0.15). ACI = autologous chondrocyte implantation; CI = confidence interval.

a Nagelkerke’s *R*^2^ = 0.345 for the full model.

b Nagelkerke’s *R*^2^ = 0.339 for the final model.

* Factor significantly influences risk of arthroplasty (*P* < 0.05).

This combination of 6 parameters (**Table 8**) represented the best-fit reduced model for predicting survival of ACI repair (with arthroplasty as the endpoint) and was used to construct an index of risk of arthroplasty post-ACI treatment (the Oswestry Risk of Knee Arthroplasty index [ORKA-1 index]). First, the coefficients (in **Table 8**) were shrunk to minimize overfitting.^29^ Based on the adjusted coefficients of the optimized Cox model (**Table 9**), the “risk index” (RI) was constructed (RI = 0.054 × A + 0.9 × F + 0.9 × NoD + 1.3 × P + MPO − 0.028 × PoL; where A = age, F = female, NoD = number of defects, P = patellar defect, MPO = multiple previous operations, PoL = pre-operative Lysholm score). An individual risk index was calculated (rounded to the nearest integer) for each patient in the study. Values for the whole group ranged from −1 to 8; patients with consecutive risk indices were grouped together when no statistically significant difference between them was found (**Table 10**).

**Table 9.** Factors and Their Multipliers in the Prognostic Model.

Factor	Multiplier
Age (A)	0.054
Female (F; score 1 if female, otherwise 0)	0.9
No. of defects (NoD)	0.9
Patellar defect (P; score 1 if patellar defect, otherwise 0)	1.3
Multiple previous operations (MPO; score 1 if MPO, otherwise 0)	1
Preoperative Lysholm score (PoL)	−0.028

Multipliers based on penalized cross-validated Cox regression model.

**Table 10.** Risk Groups for Survival to Knee Arthroplasty.

Risk Index Value (Rounded)	No. of Patients in Study	Risk Group
−1, 0, 1, 2	66	1
3, 4	71	2
5	9	3
6	3	4
8	1	5

Patients within the same risk group have survival probabilities that could not be distinguished using a log-rank test. In our study, no patients with a score of 7 was present.

**Figure 4.**
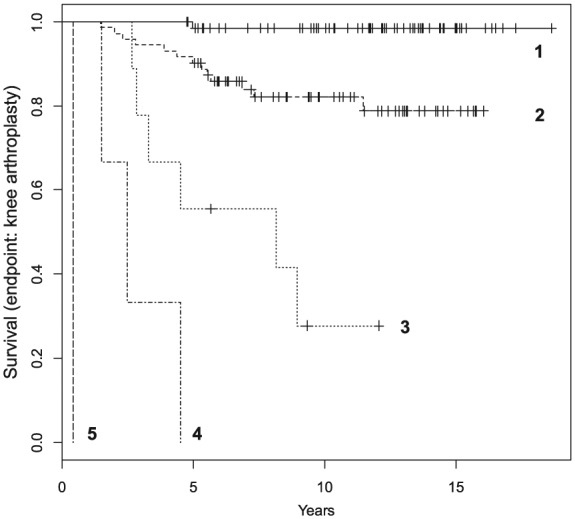
Survival of autologous chondrocyte implantation repair with knee arthroplasty as the endpoint for patients in this study belonging to the 5 identified risk groups (1-5).

To use the ORKA-1 index a patient’s risk index would first be calculated using this formula to find the patient’s designated risk group (groups 1-5). The chance of that patient requiring an arthroplasty in a period of 15 years after ACI procedure can then be estimated using **Figure 4**. For example, a patient in risk group 1 has approximately 1% chance of needing arthroplasty at 10 years after ACI, whereas a patient in risk group 3 has approximately a 70% chance.

Collagen membrane was used to cover the defects in 71 patients (42%) and a periosteal patch used for 97 patients (57%). One patient with multiple defects received both periosteum and collagen membrane; patch details for one patient were unavailable. One year post-ACI, 74% (*n* = 125) underwent a knee arthroscopy; of these, 80 (64%) had received periosteum, 44 (35%) a collagen membrane, and 1 (1%) patient had received both patch types. Hypertrophic tissue was trimmed in 55 (44%) patients who had an arthroscopy, 38/80 (48%) of patients with periosteum, and 17/40 (43%) of patients with a collagen membrane (difference not significant, chi-squared test, *P* = 0.57).

## Discussion

ACI has been used clinically for over 20 years to treat focal cartilage defects in articular joints, and it is clear that not all patients benefit. Identifying and understanding why ACI is not always successful could aid selecting the appropriate patient for this treatment in the future, improving outcomes and delaying or obviating the need for arthroplasty.

In our study of 170 patients (followed for up to 19 years), 40 (23.5%) patients had further surgery at a mean of 11 years post-ACI. This number did not include follow-up arthroscopies as in our center we have a long history of undertaking these as part of routine assessment and to provide prognostic advice,^30^ although now they are only undertaken as part of a clinical trial. Although slightly higher than the percentage of patients reporting surgical re-intervention after ACI in some studies,^31-34^ we followed our patients for longer than most. A large study of >300 patients, comparing 3 different ACI techniques over 4.5 years, reported that 16.8% of patients needed re-intervention,^34^ but did not specify the nature of surgery. The same group went on to report further that in a group of 413 patients, 21.3% had undergone re-intervention within 5 years following ACI.^35^ Other studies have included surgical re-intervention within their broad definition of “treatment failure.”^4,6^ One study reported an overall failure rate of 16% (at a mean of 7.4 years post-ACI), ranging from 11% in patients with isolated condylar lesions to 24% with patellar lesions.^6^ Another large study evaluating the functional outcomes in >800 patients treated with ACI/MACI reported an overall failure of 22% at 5 years and 49% beyond 10 years.^4^ The latter study included “return to baseline” in its definition of failure. Our finding that at 8 years 50% of patients and at 10 years 61% of patients recorded that their clinical function had returned to baseline levels points to a similar result. When comparing these 2 studies, it is important to keep in mind that our patients return a score annually, which increases the likelihood that a return of the Lysholm score to its baseline level is detected. It is also important to realize that due to ageing the natural trend of functional scores is downwards, something both studies did not account for when regarding “return to baseline” as a failure. A better insight in this natural trend is needed for a more realistic implementation of “return to baseline” in future studies.

A study of 40 patients treated with ACI (for single condylar defects) reported reoperation rates of 5% at 2 years and 22.5% at 5 years, with 2.5% undergoing arthroplasty.^32,33^ In a longer-term durability study (over 10 years), 4/72 patients (6%) had arthroplasty at 5-year follow-up and 6/72 patients (8%) at 10 years.^36^ The incidence of arthroplasty in our study (10% at 5 years and 14% at 10 years) was higher than that reported in these other studies, but set against the large proportion of patients with multiple defects and patellar defects, both predicted to increase the risk of arthroplasty, this is perhaps not surprising.

Surgical re-intervention (or revision surgery) is often used as failure-endpoint to evaluate various therapies.^10-12^ However, not all complications following ACI requiring re-intervention should necessarily be perceived as indicating treatment failure. Some techniques such as debridement of a hypertrophic transplant have improved function and reduced pain.^12^ Higher age at ACI was identified as a risk factor for re-intervention and arthroplasty in our study, but not for a return of clinical function to that recorded at baseline. Its role in predicting arthroplasty is not surprising since osteoarthritis is an age-associated disease.^37^ Most surgeons will not advise arthroplasty for the younger patient due to the finite lifespan of the prosthesis and subsequent complications. Patients having arthroplasty before age 50 are likely to outlast their implant and require revision surgery.^16^ Therefore, if ACI can delay having arthroplasty before that age it should be considered a successful procedure, even if the joint eventually does require a prosthetic replacement.

Patients usually present in our clinic with complex pathologies since we are based in a secondary/tertiary referral center. An example is cartilage lesions of the patellofemoral joint; many authors agree that these defects are the most difficult to treat.^1,4,38^ Concomitant surgeries to correct knee deformities with ligament instabilities or skeletal malalignment are now commonly performed with ACI (e.g., in 41% of our patients; **Table 1**). Increased success rates and benefits of combining treatments, such as realignment procedures with cell therapy techniques, have been reported,^6,39-41^ demonstrating the importance of treating coexisting abnormalities if indicated.^42^ However, treatment of cartilage defects prior to ACI appears to influence outcome adversely regardless of the failure definition. At least 3 other studies report an association between increased failure risk and previous treatment,^35,43^ in particular marrow stimulation techniques.^12^ In our own study, patients undergoing multiple operations before ACI were at higher risk of arthroplasty.

In our study, patients requiring arthroplasty post-ACI were more likely to be female. A trial of ChondroCelect versus microfracture, similarly found higher failure rates for women in both treatment arms.^44^ Female gender has also been associated with a greater revision rate after ACI in another study.^35^ Hormonal influences on chondrogenic differentiation, or differences in knee joint laxity, muscle strength, cartilage thickness, defect locations, and work-related or other activities of daily living between the sexes may contribute to this difference in failure risk after ACI.^45-49^ Our study also suggests that this higher rate of arthroplasty in females was not related to clinical function returning to baseline levels, because gender did not affect this reduction in Lysholm score. More subtle differences between the genders, such as in being offered the opportunity of a knee replacement or in accepting knee replacement, may thus also play a role. It is perhaps not surprising that gender is found to influence ACI outcome as differences between males and females have been noted for many years in the prevalence, incidence, and severity of joint disorders, including osteoarthritis.^31,50^

Various prognostic scoring systems are used in medicine, for example, predicting the incidence of knee osteoarthritis,^51,52^ estimating life expectancy in cancer patients,^53-56^ and assessing stroke risk,^56,57^ but to our knowledge no other predictive tool currently estimates time to requiring arthroplasty after ACI. We have developed the ORKA-1 index to estimate the long-term hazard of arthroplasty after ACI to be used before ACI treatment. We found that the preoperative Lysholm score was an important risk factor for the need for further surgery and arthroplasty after ACI. Improvement in Lysholm score generally occurs within the first 2 years after ACI,^33,58^ following which the Lysholm score remains constant.^58^ Improvement in knee function can be maintained for over 10 years after treatment.^6^ We found that >70% of patients showed an improvement in clinical outcome at 1 year, supporting previous findings.^58^ The group that went on to have arthroplasty were quite different, with no significant increase in 1-year Lysholm score, which then decreased rapidly with time post-ACI. We have included many parameters that are regularly recorded in our center, but recognize that further parameters might also prove relevant. This index has been developed with first- and second-generation ACI, but it should be possible and relevant to be used as a predictive factor for all biological or cell therapy treatment of chondral and osteochondral defects. There is certainly a need now for the ORKA-1 to be validated in a second population of ACI-treated patients. The ORKA-1 index is now being used in the preoperative clinic in our center, which will likely provide this opportunity, but ideally it will also be tested and validated in other centers with different patient populations and surgeons. We also envisage that it will be modified and improved in the future, as well as simplifying its use. For example, it lends itself to being developed into a very easy to use “app” for use on smart phones and tablets in any location in the clinic.

As with all studies, the present one has some limitations. First, it is a single center study with the majority of patients being treated by one surgeon, so has the potential to be influenced by selection bias. On the other hand, it is based on a heterogeneous group of patients with cartilage defects representing a mixed group in terms of age, size of defect, anatomical location of defect, co-injuries, and previous surgery, which will help generalization to new patient cohorts. Our broad study inclusion criteria compared with those of typical clinical trials (e.g., Knutsen *et al*.^32^), makes our study cohort perhaps more representative of patients with a more severe and complex disease. Not only have randomized clinical trials usually a more narrowly defined study population but they also have potentially high expectations.^59^ Our patient group is perhaps more representative of the general population with cartilage injuries or early osteoarthritis.^60^ Unfortunately, the data set is not 100% complete for all patients treated in our center with some values missing for 30 of the 170 patient group. While this is not perfect, and undoubtedly would have been much smaller if the study had been a proper clinical trial, the missing data only represent aspects of the patients and is probably missing completely at random. The total number of patients is quite high and covers a long time span compared to most other studies reported in the literature. A final limitation is that, although we have performed an internal validation of the ORKA index using a cross-validation and shrinking procedure to guard against overoptimism,^26^ our predictive model still requires external validation.

In conclusion, the incidence of further surgery post-ACI treatment within our study over an average of 11 years was 23.5%. The incidence of joint failure (or the requirement for arthroplasty) was 15%, more commonly in females. Age, gender, defect number and location, previous surgery, and preoperative Lysholm score are important factors to consider when deciding which patients would benefit most from ACI for cartilage defects in the knee. We propose that combining these predictors into the ORKA index to estimate the likely time to arthroplasty will help both the patient and surgeon decide whether to proceed with ACI as an appropriate surgical option for the treatment of cartilage defects.
